# Analysis of Public Datasets for Wearable Fall Detection Systems

**DOI:** 10.3390/s17071513

**Published:** 2017-06-27

**Authors:** Eduardo Casilari, José-Antonio Santoyo-Ramón, José-Manuel Cano-García

**Affiliations:** Departamento de Tecnología Electrónica, Universidad de Málaga, ETSI Telecomunicación, 29071 Málaga, Spain; jasantoyo@uma.es (J.-A.S.-R.); cano@dte.uma.es (J.-M.C.-G.)

**Keywords:** fall detection, dataset, accelerometer, wearable, smartphone, mHealth

## Abstract

Due to the boom of wireless handheld devices such as smartwatches and smartphones, wearable Fall Detection Systems (FDSs) have become a major focus of attention among the research community during the last years. The effectiveness of a wearable FDS must be contrasted against a wide variety of measurements obtained from inertial sensors during the occurrence of falls and Activities of Daily Living (ADLs). In this regard, the access to public databases constitutes the basis for an open and systematic assessment of fall detection techniques. This paper reviews and appraises twelve existing available data repositories containing measurements of ADLs and emulated falls envisaged for the evaluation of fall detection algorithms in wearable FDSs. The analysis of the found datasets is performed in a comprehensive way, taking into account the multiple factors involved in the definition of the testbeds deployed for the generation of the mobility samples. The study of the traces brings to light the lack of a common experimental benchmarking procedure and, consequently, the large heterogeneity of the datasets from a number of perspectives (length and number of samples, typology of the emulated falls and ADLs, characteristics of the test subjects, features and positions of the sensors, etc.). Concerning this, the statistical analysis of the samples reveals the impact of the sensor range on the reliability of the traces. In addition, the study evidences the importance of the selection of the ADLs and the need of categorizing the ADLs depending on the intensity of the movements in order to evaluate the capability of a certain detection algorithm to discriminate falls from ADLs.

## 1. Introduction

Falls are a major source of loss of autonomy, deaths and injuries among the elderly and have a remarkable impact on the costs of national health systems. According to World Health Organization [1], 28–35% of the population over 64 experience at least one fall every year, while fall-related injuries and hospitalisation rates are expected to increase on average by 2% per year until 2030 [2]. Morbidity and mortality provoked by falls are closely connected to the rapidness of the medical response and first aid treatment after the incident [3]. Consequently, the analysis of cost-effective and automatic Fall Detection Systems (FDSs) has become a relevant research topic during the last years.

A FDS can be regarded as a binary classification system envisioned for discriminating fall events from any other movement of the user (the so-called Activities of Daily Living or ADLs). One of the key issues in the investigation on fall detection systems is the lack of a consensus about the exact procedure by which FDSs should be evaluated. Due to the inherent difficulties of testing a FDS with actual body falls (especially falls of older people), only a reduced number of works have examined the effectiveness of a FDS with real-world falls. In [4,5,6,7,8] a small group of older people (from 3 to 20 persons) were tracked during some weeks to check the accuracy of the proposed FDS. In all cases, very few falls (or even no falls) occurred during the monitoring period, so just the capacity of the system to avoid false alarms could be actually assessed. In [9] the performance of different fall detection algorithms was benchmarked when they are applied to a database of 29 real falls obtained by monitoring nine highly impaired Parkinson’s patients (a population group with very specific psychomotor disorders). In any case, none of these datasets obtained through long-term tracking of patients has been released.

The FAll Repository for the design of Smart and sElf-adaptive Environments prolonging INdependent livinG (FARSEEING) project collected a real-world fall repository which gathers a collaborative database of more than 300 real-world fall events recorded with different inertial sensors in several studies [10]. A dataset (of only 20 selected fall events) is available for researchers on request. This FARSEEING project also generated a list of recommendations (drafted by a multi-disciplinary panel of experts) about the characteristics (fall definition, sensor configuration, variables to represent the monitored signals, etc.) that a benchmarking dataset of falls should present.

In the absence of clinical databases of actual falls [11], most research studies utilize mimicked falls, normally emulated by a group of volunteers, who also execute a certain set of ADLs to check the effectiveness of the detection process. The lack of a standardized framework about the evaluation of FDSs is reflected in the inexistence of a common reference dataset massively employed by the research community to compare the performance of the proposals existing in the literature. As a matter of fact, in the vast majority of studies dealing with fall detection algorithms, authors create their validation samples by defining their own testbed with their particular group of experimental subjects and arbitrarily predefined typologies of falls and ADLs. These generated datasets are rarely published or made available. Therefore, the reproducibility of the tests and the cross-comparison with other algorithms is very difficult.

In order to fill this knowledge gap, different datasets have been recently produced and released by different groups devoted to the research on fall detection. In this paper, we review and analyze thoroughly those existing datasets that have been created to evaluate wearable FDSs.

This paper is organized as it follows: after the introduction in Section 1, Section 2 presents a basic taxonomy of fall detection systems. This classification will allow identifying which datasets will be under study. Other databases associated to the research on fall detection and movement recognitions will be also summarily reviewed in this section. Section 3 identifies the selected datasets, summarizing the bibliographic procedure that was followed during the selection. Section 4, Section 5 and Section 6 describe in detail the properties of the traces under analysis (characteristics of the experimental users, employed sensors, etc.) while Section 7 offers a detailed statistical comparison of the datasets. Finally, Section 8 recaps the main conclusions of the paper.

## 2. Types of Fall Detection Systems: Review of Other Datasets

Depending on the nature of the employed sensors, Fall Detection Systems can be classified into two generic groups [11]. Firstly, Context-Aware Systems (CAS) are based on sensors located in the environment around the user to be supervised. Thus, CAS solutions typically integrate vision-based and ambient-based systems, including cameras, microphones, vibration sensors, etc., which are deployed on a specific “tracking zone” (e.g., a domestic room) where the user will be monitored. Due to this constrained supervision area, the vulnerability of CAS detection decisions to external events (such as tumbling objects, unexpected noises or changes of the illumination levels) and the high costs related to the installation and maintenance of CAS-type detectors, wearable FDSs have received more attention in recent years. Wearable systems employ accelerometers (and other mobility sensors) which are attached to the clothes or transported by the patients as personal garments. Wearable FDSs directly estimate the user mobility without altering his/her sense of privacy and with independence of the particularities of the environment. Unlike CAS architectures, given that wearable FDS can be easily provided with communication interfaces (e.g., 3G/4G connection), user can be monitored almost ubiquitously at a low cost. Smartphone-based FDS are a particular example of wearable FDS which has been very studied by the literature. Smartphone-based architectures may clearly benefit from the fact that a single commercial device natively integrates an inertial measurement unit (IMU) (including and accelerometer, a gyroscope and in some cases, a magnetometer) while supporting multi-interface wireless communications (Wi-Fi, 3G/4G, Bluetooth).

In any case, automatic detection of falls using artificial vision can be regarded as a particular case of human activities recognition. Concerning this, there are some available databases intended for the research on the visual recognition of human activities and body silhouette tracking, which also include video sequences or images of emulated falls (as another activity to be discriminated). We can find examples of these databases offered by research centers such as CNRS at Dijon (France) in [12], the Université de Montréal (Canada) in [13] the University of Leuven (Belgium) in [14], the University of Texas at Austin (U.S.) in [15] or the Universidad Politècnica de Valencia (Spain) in [16]. These datasets are out of the scope of this paper (see [17] for an extensive review on this matter) although we do consider those databases that were conceived to test hybrid CAS-type and wearable FDS, i.e., systems that make their detection decision from the combination of image-based and accelerometer-based techniques.

We did not take into consideration either those mobility databases obtained with wearable IMUs that do not include falls among the activities performed by the monitored users (i.e., datasets that were not conceived to evaluate FDSs). In order to obtain the samples, these benchmark databases follow the same general methodology of the databases that will be studied in this article. So, the data are collected from a group of experimental subjects who transport one or several mobility sensors (mainly accelerometers) attached to different parts of the human body while executing ADLs (predefined or not). Among these databases that are exclusively oriented to test activity classification systems, we can mention the WISDM [18], DaLiAc [19], SCUT-NAA datasets [20] or the ‘Human Activity Recognition using Smartphones’ dataset [21] as well as those offered by the web page www.ActivityNet.org [22] of the Pattern Recognition Lab in the University of Nuremberg-Erlangen (Germany) or in the UCI Machine Learning Repository [23]. In addition, there are platforms, such as PiLR EMA [24], which have been developed to facilitate data collection about the weight and physical activities of study participants through the use of a smartphone application. In the context of CrowdSignals.io (a campaign to build massive and comprehensive databases from the sensors embedded in smartphones and smartwatches) the sample datasets provided by Algosnap in [25] include the data captured by 40 sensors and data sources from Android smartphones, smartwatches and Microsoft Band 2 devices. The data were collected by two participants wearing these devices during their daily life. The time intervals in which the activities (washing hands, driving, eating, standing, etc.) are carried out by the subjects are manually labeled in a specific document that accompanies the traces.

## 3. Search Methodology and Basic Information of the Existing Datasets

The search for publicly available datasets was carried out through a systematic exploration of most common databases of peer-reviewed research sources (IEEE Explorer, Scopus, Ovid SP and Cochrane library). The examination of the related literature was limited to publications in English and mainly based on text string searches in the title, abstract and keywords of the papers. The search terms were “fall detection” combined with “dataset”, “repository” or “database”. The use of these citation databases was complemented by other academic reference managers (such as Mendeley or Google Scholar) and other basic web search engines (e.g., Google). In addition, cross reference search was done in the retrieved manuscripts. The exploration was considered to be finished in April 2017. As aforementioned, during the bibliographic search we only considered those samples obtained for the research on wearable FDSs.

At the end of this search, we found 12 publicly available datasets aimed at the research on wearable FDS. Koshmak et al. in [26] and Igual et al. in [27] compared the application of supervised feature learning techniques to three of these datasets (DLR, tFall and MobiFall). In the study by Koshmak, the analysis was extended to a fourth dataset, derived from the EU-founded GiraffPlus Project [28]. However, as far as we know, this trace is not available in the Web. In both studies, important dissimilarities among the datasets were detected. In [27], authors found that the performance of the detection algorithms may strongly depend on the employed samples, showing the difficulties of generalizing the capability of a certain FDS when it is only checked against a single dataset.

The name, authors, institution, bibliographic reference and release year of the traces are presented in Table 1 (one of these datasets, MobiAct, is actually an extended version of another one, MobiFall, as long as it incorporates a new group of traces). Investigation on wearable FDSs emerged as a popular research topic since 2010. This fact is evidenced in the fact that most public datasets have been generated and released since 2014.

Table 2 includes the URLs (active in April 2017) from which the databases can be accessed and the format in which the traces are codified. In this regard, the table shows the diversity of employed formats, ranging from a collection of simple text files to SQL databases. Nevertheless, traces in several datasets are stored in plain text by means of comma-separated values (CSV), a flexible tabular format that can be easily processed through different software environments such as spreadsheet programs or high level mathematical tools (such as Matlab (The MathWorks, Inc., Natick, MA, USA)).

As it refers to the way in which datasets are organized, in most cases, an individual file is generated whenever an experimental subject carries out a certain activity (fall or ADL). Thus most datasets consist of a long list of log files, each of which contains the monitored measurements of a specific movement executed by a particular subject.

## 4. Analysis of the Testbed: Experimental Subjects and Scenarios

All the found datasets were generated by a set of volunteers that were specifically recruited for that purpose. Table 3 shows the number and the gender of the subjects for the different databases. The table also includes the range, mean value (μ) and the standard deviation (σ) of three variables which are usually described in all testbeds with experimental subjects: age, weight and height. In some datasets (e.g., tFall) the associated documentation describes the global parameters of the group but not the individual characteristics of each subject.

The data in the table show the great variety of criteria with which these groups of volunteers are selected. Except for SisFall and UniMiB SHAR datasets, the predominance of the number of men against women in most databases is noteworthy. As it could be expected, the groups of volunteers are clearly formed by young adults (with a mean age almost always below 30 years). In the few cases in which adults over 50 years old participated in the experiments, for the sake of safety, they did not emulate falls and their actions were often limited to certain ADLs that did not pose any risk. Some testbeds enrolled people with special physical abilities to emulate a fall. For example, a specialist in judo and aikido aged over 50 participated in the generation of SisFall dataset while the Graz samples were also captured from the parallel movements of five martial artists.

In all the associated documents describing the datasets, authors indicate that the subjects are in good health or at least, they do not comment any pathology or disability that could affect their movements. In this sense, it is still under discussion if the signals captured from healthy young adults who emulate a fall (or athletes who are skilled to cushion hits provoked by falls) can be extrapolated to the mobility patterns that older people actually present during a fall [33]. Authors in [41] have shown that the effectiveness of mobility pattern classifiers clearly plummets when the recognition of the dynamics of a certain individual is founded on other subject’s gait. On the contrary, Kangas et al. compared in [5] the acceleration signals of intentional falls and those captured from actual falls suffered by long-term monitored elderly, concluding that they exhibit a similar profile.

Anyhow, although the age, weight, height and gender of the employed experimental population are normally provided in most studies on FDS, these parameters are not employed to discriminate the validation samples during the evaluation phase. Future research studies should take advantage of the existence of these datasets to investigate the influence of these parameters on the detection process.

Another interesting point, described in Table 4, is the conditions and scenario in which the movements of the volunteers were developed. The table also includes information on another important aspect, informing if the datasets are accompanied by video clips and images that allow to portray the testbed and the nature of the movements beyond a simple textual description (in fact, in some datasets, the scenario commented in Table 4 was deduced by observing the pictures in the documentation and/or the video sequences included in the web site). As it can be observed from the table, in most cases, both falls and ADLs were simulated and scheduled according to predefined types. Only in two datasets (DLR and tFall) ADLs were captured in ‘natural’ or ‘semi-natural’ conditions by tracking volunteers during their daily living activities (without responding to pre-established rules or patterns). In point of fact, these datasets of unplanned ADLs are those that were obtained in the most realistic environments (including outdoor spaces such as a forest or a bus stop). In the other datasets, the experiments took place mostly in gyms and laboratories. In the case of UMAFall and URFall, domestic environments were also utilized.

Regarding the scenario, another controversial issue in the definition of testbeds for FDS is the use of cushioning material and/or safety elements (knee-pads, helmets, etc.) to protect the experimental subjects during the falls. Table 4 shows that most datasets utilize mattresses or foam pads to cushion the falls. Tumbling onto a pad may introduce another important divergence in the captured acceleration signals when compared with those caused by undesired impacts against the ground. Contrariwise, if these protective elements are not employed, fear of getting injured could alter the behavior of the subjects when they execute the falls. There is not much literature about these issues. The impact of all these aspects is still open to further discussion and should be addressed with specific experiments.

## 5. Number, Duration and Typology of the Movements

As it is displayed in Table 5, almost all datasets were created by the planned and repeated execution of a predefined series of types of activities (ADLs or falls). Only in the case of the tFall dataset, ADLs did not follow an “a-priori” typology and were recorded by monitoring subjects freely moving during several days in their daily lives.

In order to generate the traces in many datasets, sample recording was discontinued whenever a certain activity was accomplished by the corresponding subject. Consequently, a file was produced for every activity and trial. Nevertheless, in some datasets (as in Cogent Labs, Graz and UniMiB SHAR), subjects performed a series of predetermined activities sequentially, without being interrupted. The resulting sample was then analyzed and manually annotated to enter the type of activity that the subject was executing at any time. For the UniMiB SHAR dataset, the subjects were asked to clap their hands before and after executing each activity so that the audio signal that was also recorded during the movements could help in this annotation or ’labeling’ process. In the case of the traces from BMI Lab (Mobiact & MobiFall), the subjects emulated the falls in two different ways: with and without a long interval of lying, which was aimed at replicating the period in which a person may remain unconscious after collapsing on the ground.

Table 5 shows that the duration of the samples is variable in the majority of the cases, oscillating from less than 1 s to several minutes. In UMAFall dataset, a common fixed period (15 s) is set to emulate each activity and record all the experiments. All the acceleration traces in UniMiB SHAR were filtered and just include 51 samples (1 s) centered around the detected peak of the acceleration magnitude. Thus, the pre-fall and post-fall periods (which are considered in some detection algorithms) cannot be analyzed. A similar policy was followed in the tFall dataset. To obtain the ADLs, users were constantly monitored but entries only include the information of a time window of 6 s around the peak in the acceleration magnitude (on condition that this peak was detected to exceed a certain ‘recording’ threshold of 1.5 g). For the fall simulation, the same interval of 6 s was selected.

Table 5 also illustrates the large variety in the length of the datasets (global number of samples describing activities—falls or ADLs—). Detection algorithms are evaluated in terms of typical performance metrics such as the sensitivity or the specificity, which are estimated from a ratio depending on the number of false and true positives and negatives that are achieved when the algorithm is applied to a certain set of samples. Accordingly, the confidence interval and the reliability of these metrics strongly depend on the number of samples. Thus, length is one of the most strategic parameters to determine the interest of a dataset. In this regard, the largest databases correspond to tFall, UniMiB SHAR and MobiAct sets (with more than 2500 samples). On the contrary, the traces TST, UR and Gravity Project dataset include fewer than 300 samples. The number of falls should also be taken into account for the selection. Except for TST dataset, there is always an asymmetry between the number of recorded ADLs and falls. This asymmetry is especially remarkable in the DLR dataset, which only encompasses 56 falls (and 961 ADLs).

Another relevant selection criterion for the datasets may lie in the variety of ADL and fall types that were considered to generate the records. Table 6 details the typologies used for the ADLs and falls in the analyzed datasets. In the tFall dataset, ADLs are neither annotated nor typified as they are captured by tracking users in real-life conditions during several days.

There are works in the literature that have systematically typified the falls. In [42] Yu classified typical falls among elderly into three groups: falls from sleeping, from sitting and from standing. Based on that typology, Abbate et al. [43] suggested a set of 20 categories of falls and 16 ADLs as a benchmarking scenario for FDSs. However, although there are activities (such as sitting) that are present in all datasets, Table 6 reveals the lack of a common strategy in the literature for setting the number and types of ADLs.

It should be noted, though, that in no dataset did the authors organize the employed types of ADLs according to the actual mobility they demand. Thus, there is not a previous distinction between those activities that hardly require any movement (such as standing) and those that even entails some significant physical effort (such as running). In this sense, we propose to classify the ADLs of the datasets into three large subcategories depending on the degree of movement that is needed. Hence, Table 6 categorizes the registered ADLs into three great groups: basic movements (such as sitting, getting up, lying down, turning or getting into a car), standard “everyday” movements that require a higher mobility or a certain physical effort (walking, climbing stairs, bending, tying shoes, etc.) and, finally, sporting activities (jumping, running, jogging, hopping). As it will be observed in Section 7, the dynamics and the behavior of the acceleration signals captured from the movements of these three subcategories may strongly differ. We consider that this classification of the ADLs is relevant because its use for the evaluation of a FDS can help to characterize the efficiency of a fall detector depending on its application scenario and on the target public for whom the system is intended. So, if the FDS is planned to be employed with elderly people with severe mobility problems, FDS may only require to be tested with ADLs from the first group. On the contrary, the three subcategories should be contemplated if the FDS is aimed at detecting falls of other population groups (e.g., healthy active elderly, aerial technicians, firefighters, mountain climbers, athletes, etc.).

Regarding falls, the criterion that is massively considered to typify these movements is the direction of the body when a fall occurs (forwards, backwards, rightwards, leftwards or syncope). From these large categories of falls, variations are defined depending either on the activity before falling (walking, sitting, etc.) or on the final position of the subject after the collapse. This “directional” definition of the movements allows a more objective description of the falls while it eases their replication. Only the Graz database defines the fall types according to more subjective criteria, associated not to the direction of the falls but to the movements that cause them (stumbling, slipping, sliding, getting unconscious). In any case, the inclusion of video clips in the datasets is very helpful for the identification of the employed typology because they offer a real idea of the activities that have been actually executed.

A special group of ADLs (which we have not considered within any of the three defined subcategories) are the “near-falls”, which are only present in the samples of Cogent Lab and SisFall. These ADLs can be used to refine the assessment of the discriminatory capacity of FDS. However, provided that both falls and near-falls are expected to be rare events, the fact that a FDS produces a false positive in the occurrence of a near-fall does not imply the same underperformance as in the case of a confusion with a much more basic and frequent activity. In any case, the credibility of these recorded activities depends greatly on the ability of volunteers to emulate movements such as slips or tripping that do not result in a fall.

Except for the Cogent dataset, where there are falls caused by unexpectedly pushing a subject whose eyes were previously blindfolded, the falls in all datasets correspond to emulations. In this regard, the legitimacy of evaluating FDSs with intentional falls is another topic under permanent discussion. It has been stated [44] that real-life falls follow a faster and more irregular mobility pattern than those which are mimicked. This discrepancy is also highlighted by Klenk et al. in [45] after analyzing the dynamics of a small set of falls experienced by older people.

The study in [9] shows in turn that the efficacy of FDSs deteriorates when applied to actual falls. Conversely, Kangas [46] examines the acceleration data of falls among aged people and concludes that real-world falls present a similar behavior to that exhibited by falls that are mimicked in a laboratory. This debate is beyond the scope of this paper. Anyway, the lack of a public database with a representative number of mobility samples captured from actual falls obliges to utilize the databases with emulated movements.

## 6. Number, Characteristics and Position of the Sensors

As aforementioned, wearable FDSs typically consist of a certain number of transportable “sensing points” meant for characterizing the user’s movements. Every sensing point may in turn contain several sensors. Table 7 informs about the number and position of the sensing points employed for the generation of the analyzed datasets as well as about the sensors utilized by every sensing point.

As it can be observed in the table, most measurement systems are based on a single sensing point, normally located—for all experiments—in the same position. The samples of TST and Cogent Lab (as well as some traces of the Gravity Project) incorporate the measurements recorded by two sensing points, while UMAFall samples were obtained through a network of five sensors located on five parts of the body. For both Cogent Lab and UMAFall datasets the system was deployed through a Bluetooth wireless network in which the sensing points transmit the captured signals to a central point (a computer in the case of Cogent and a smartphone, which also performed as a fifth sensing point, for UMAFall). The authors of the TST dataset do not detail in the attached documentation whether the used sensors formed a network or if they stored the data locally.

The use of datasets with more than one measurement point fosters the study of another research topic in the investigation of transportable monitoring systems: the selection of the optimal position of the sensors and the advantages of combining information from more than one measurement point. The placement of the sensors for the discrimination of everyday activities has been analyzed in [47] while the papers [36,48,49] studied the advantages of combining the measurements of a smartwatch and a smartphone to improve the effectiveness of a fall detector app. The goal of UMAFall dataset is to ease a systematic research of the impact of the position on the efficacy of the detection process.

In the case of systems with a single sensing point, the positions where the efficacy of a wearable FDS is optimized have been claimed to be the chest and the waist [50]. This fact can be explained by their position close to the human body’s center of mass. Conversely, pockets offer users an easygoing and comfortable way to wear the sensors. However, the use of loose pockets (or even hand bags) to transport the sensing point may clearly provoke a reduction in the attachment to the body. Authors in [51] showed that the performance of a smartphone-based detector is noticeable affected when the device shifts within the pocket.

Consequently, a trade-off between the efficacy and the ergonomics of the system must be always achieved. In this respect, the chest may be an ‘unnatural’ position for a sensor (especially if a smartphone is employed as the sensing point) as long as attaching a sensor to the user’s chest may cause some discomfort. The location on the waist may offer a better alternative if the user wears a belt where the sensors can be easily adapted. As Table 7 reveals, the subjects in all the testbeds at least employ either the waist or a pocket to carry the sensors.

As it refers to the employed types of sensors, all the testbeds utilize accelerometers, since the accelerometry signals are by far the most common physical variables used by fall detection algorithms in wearable FDSs. What is more, the monitoring unit in the SisFall dataset incorporates two different accelerometers at the same measurement point (located at the waist). Besides, six datasets also make use of the signals measured by a gyroscope to include information about the angular velocity and the orientation of the movements. Finally, although magnetometer signals are not considered in most literature about detection algorithms, there are two databases (Cogent and UMAFall) that include the records obtained by the magnetometers embedded in the sensing points. So, every measurement in the traces normally consists of a timestamp followed by the three components of the acceleration and (if available) the angular velocity and the magnetic field vectors. In the Gravity Project dataset, the module of the acceleration vector and the vertical acceleration (instead of the three acceleration coordinates) are recorded.

The measurements employed in studies on wearable FDSs are typically captured by an IMU, a MEMS device which integrates in a single component an accelerometer, a gyroscope and, in some models, a magnetometer. Practically all current smartphones embed an IMU so they have been considered in many works proposing wearable fall detectors.

Table 8 shows that five (MobiFall Dataset, MobiAct, tFall, Graz and UniMiB SHAR) out of the 12 analyzed datasets were obtained through a “stand-alone” smartphone-based platform, while the Gravity Project offers the signals captured in a smartwatch and a smartphone, which were both transported by the users during the experiments. Contrariwise, the measurements of five datasets (DLR, TST, UR, Cogent and SisFall) were based not on smartphones but on external sensors, including commercial IMUs (by vendors as Shimmer, x-io or Xsens) or (in the case of the SisFall dataset) a self-developed prototype of sensing mote comprising two accelerometers and a gyroscope. The testbed for the UMAFall dataset was deployed through a network of five nodes: a smartphone (acting as the master of a Bluetooth piconet) and four motes built on Texas Instruments SensorTags, which integrate InvenSense IMUs.

The use of the built-in sensors of the smartphones for applications of fall detection has also been a topic of discussion. The analysis is complicated because some vendors do not describe in much detail the features of the built-in sensors in the technical specification of the smartphones (e.g., in models by Apple the specifications of the motion coprocessors are not always disclosed). Moreover, range (and resolution) is a user-selectable parameter in many IMUs (including those integrated in smartphones), typically from ±2 g to ±16 g for accelerometers. However, this selection is not always clarified by the authors of the datasets.

The potential of smartphones to identify free falls has been studied by Vogt in [52], who concluded that smartphones can estimate free-fall time with a good degree of accuracy. In contrast, the work by Mehner in [44] states that the small range of some smartphone accelerometers are not adequate to discriminate falls. The range provided by the accelerometers in several models by Samsung (as those used in the MobiFall, MobiAct, tFall, Graz, Gravity Project or UMAFall datasets) is only ±2 g (about 19.61 m/s^2^), which can be enough to recognize the orientation of the screen but not sufficient to capture the brusque increase of the acceleration caused by the impact on the ground. On the other hand, the comparisons of the performance of built-in smartphone accelerometers and dedicate (or external) sensing devices by Mellone [53] and Albert [54] indicate that smartphones constitute a valid tool to characterize human mobility in a quantitative way.

Table 8 informs about the main characteristics (model, range and resolution) of the sensors employed for the datasets. In some cases, this information was not made explicit in the documentation and was indirectly deduced from the numerical series. In other cases, the range and/or resolution measured in the traces were not completely coherent (e.g., Graz dataset) with the values specified by the documentation or the datasheets of the IMUs. We can check this point in one of the columns of the table, which contains the most positive and negative values found in the data for each measured variable. The “saturation” of these values in some dataset clearly denotes that the range limits were reached in some samples (a fact that, for example, could distort the peak recognition of some detection algorithms). Besides, the units of the magnitudes are not clearly described for several sequences (e.g., the magnetic field in DLR dataset, or the acceleration—expressed in g or m/s^2^—in the Gravity Project dataset).

Another essential factor is the sampling frequency at which the information is captured by the sensors. Table 8 presents the sampling rate used in the datasets, which ranges from only 5 Hz (Graz dataset) to 256 Hz (UR dataset). The importance of the sampling rate in the efficacy of a FDS has been investigated by Medrano in [33] and Fudickar in [55]. This author states that a frequency of 50 Hz is sufficient to achieve a good detection performance. In any case, spectrum analysis of the signals is not carried out in the testbeds to justify the selection of a certain sampling period. Thus, the sampling rate is arbitrarily chosen and merely determined by the capacity of the sensors or by the operating system of the device. For example, Android offers four qualitative levels to set the sampling rate of the accelerometer, but the actual frequency (ranging from 7 to 200 Hz) associated to each level heavily depends on the smartphone model. So, two smartphones executing the same FDS application may exhibit different sampling rates. In addition, the sampling rate of smartphones is affected by the operation of the device and may vary during the captures (as it can be confirmed from the analysis of the datasets). In the Graz dataset, for example, five different smartphone models from three different vendors are employed, so different sampling rates, ranges and resolutions can be actually expected. Except for the gyroscope in the testbed of Mobifall dataset (which was adjusted before the experiments), sensors are always assumed to be correctly calibrated.

## 7. Analysis of the Datasets

This section presents a systematic statistical comparison of the datasets. The goal is to assess the divergences of the measurements as a function of the testbed where they were generated, but also taking into account the nature of the movements.

In order to analyze the datasets on equal conditions, we only take into consideration the measurements performed by the tri-axial accelerometers (which are the sole common sensor in all the repositories) located on a similar position: the waist (by default) or thigh (when the measurements on the waist are not available). All the numerical series obtained from the repositories were transformed into a common format (a Matlab structure array) and then post-processed with Matlab scripts.

Most threshold-based algorithms and machine learning strategies proposed in the literature on FDSs take the module of the acceleration vector (or SMV, Signal Magnitude Vector) as the reference (or even unique) parameter to be analyzed for the detection decision. Thus, we focus our analysis on this variable. For the *i*-th measurement the SMV of the acceleration measurements is defined as:

(1) $$SMV_{i}=\sqrt{A_{xi}^{2}+A_{yi}^{2}+A_{zi}^{2}}$$

where *A_xi_*, *A_yi_* and *A_zi_* represent the acceleration components in the *x*, *y*, and *z*-axis, respectively, measured by the accelerometer for the *i*-th capture.

From the sequences of *SMV_i_* we compute four basic statistics that characterize the movements of the subject:

1. Maximum value of the acceleration module, which can be estimated from the traces as:

(2) $$SMV_{max}=\underset{i\in \left\{1,2,..N\right\}}{\max}\left(SMV_{i}\right)$$

where *N* represents the total number of samples in the dataset.

This parameter is massively employed by the literature as a first recognition criterion provided that falls typically cause the occurrence of unexpected peaks of the acceleration module.

2. Minimum value of the acceleration module, which is computed as:
  (3) $$SMV_{min}=\underset{i\in \left\{1,2,..N\right\}}{\min}\left(\;SMV_{i}\right)$$

This observation of this parameter is also relevant as long as a collapse is normally preceded by a brusque drop of the acceleration. This phenomenon is due to the fact that accelerometers tend to measure zero during free-falls.

3. Maximum absolute value of the differentiated acceleration module, which can be estimated as:
  (4) $$SMV_{d_{max}}=\underset{i\in \left\{1,2,..N-1\right\}}{\max}\left|SMV_{i+1}-SMV_{i}\right|$$

This high-pass filtering of the acceleration magnitude is aimed at identifying the sudden changes experienced by the acceleration because of the impact against the floor.

4. Maximum averaged acceleration module estimated for a sliding window (*W*) of 1 s.

(5) $${\bar{SMV}}_{max}=\underset{j\in \left\{\left\lfloor \frac{N_{W}}{2}\rfloor +1,...N-\lceil \frac{N_{W}}{2}\right\rceil \right\}}{\max}\left(\frac{1}{N_{W}+1}\sum_{j-\lfloor \frac{N_{W}}{2}\rfloor }^{j+\lceil \frac{N_{W}}{2}\rceil }SMV_{i}\right);\;\mathrm{being}\;N_{W}=\lceil W\cdot r\rceil$$

where ($N_{W}$ + 1), which symbolizes the number of samples contained in the averaging window, is computed from the widow duration (*W*) and the sampling rate (*r*) at which the measurements were captured. In the expression, the operators ⌊ ⌋ and ⌈ ⌉ indicate the floor and ceiling functions, respectively. This metric, which is based on a low-pass filtering of the SVM, is intended to recognize the growth of the mean acceleration module in a time interval around the impact.

**Figure 1:**
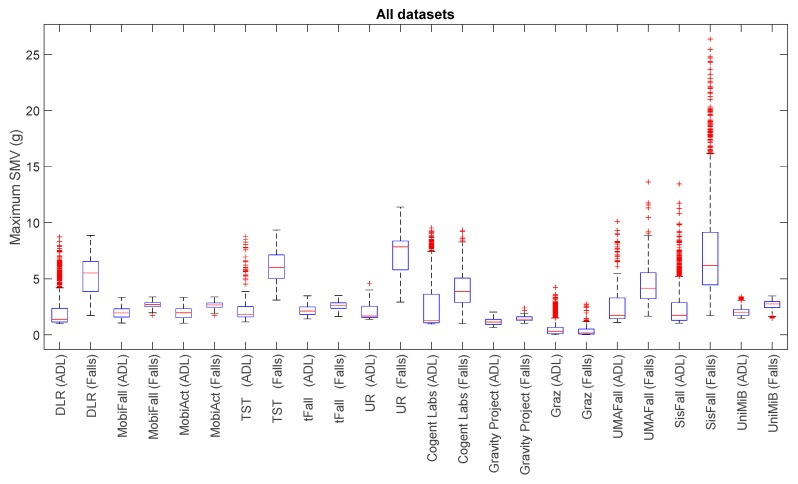
Boxplots of the of the maximum acceleration module for the falls and ADLs of all the datasets (for all the datasets only the acceleration signal at the waist or—if not available—at the thigh is considered).

**Figure 2:**
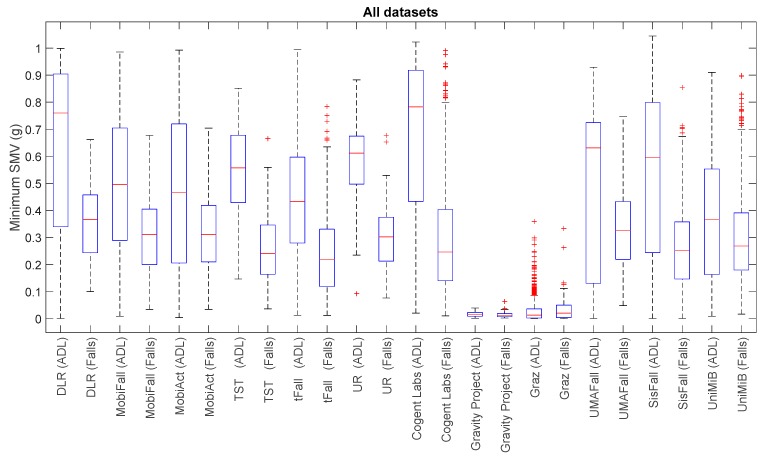
Boxplots of the of the minimum acceleration module for the falls and ADLs of all the datasets (for all the datasets only the acceleration signal at the waist or—if not available—at the thigh is considered).

**Figure 3:**
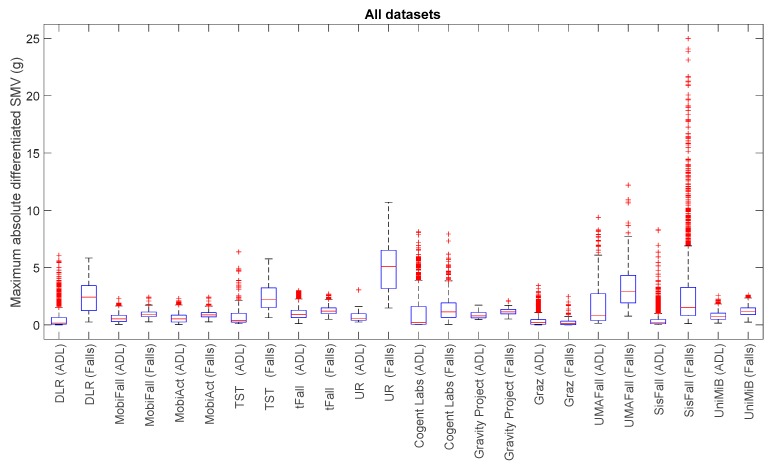
Boxplots of the maximum absolute value of the differentiated acceleration module for the falls and ADLs of all the datasets (for all the datasets only the acceleration signal at the waist—if not available—at the thigh is considered).

**Figure 4:**
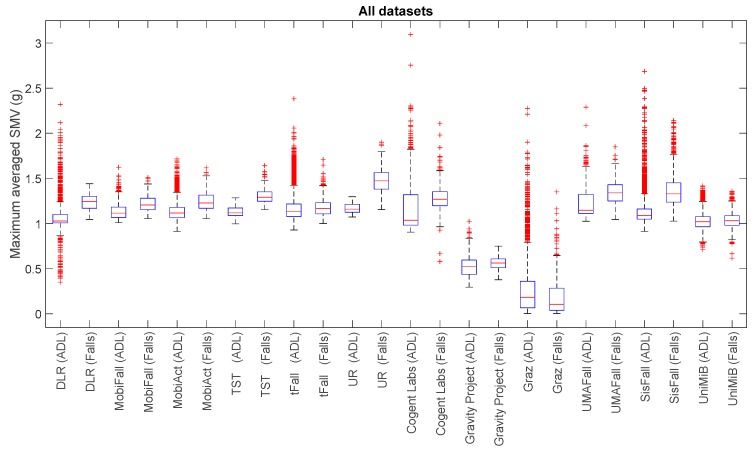
Boxplots of the maximum averaged acceleration magnitude (considering a sliding window of 1 s) for the falls and ADLs of all the datasets (for all the datasets only the acceleration signal at the waist or—if not available—at the thigh is considered).

For a visual comparison of the sample spreads of the different datasets, we utilize boxplots, a common tool for the analysis of fall detection and activity recognition systems. Figure 1, Figure 2, Figure 3 and Figure 4 depict the box-and-whisker plots of these four statistics, which have been separately computed for ADLs and falls in the 12 analyzed datasets. The central line in each box indicates the median value whereas the edges of the box represent the 25th and 75th percentiles of the metric for each dataset and movement type (ADLs or falls). The whiskers marked in dotted lines cover the most extreme data points which are not judged to be outliers. The outliers are in turn plotted as individual points (crosses) out of the interval.

The graphs display the high diversity of behaviors among the different samples. As it refers to the maximum values of SVM (Figure 1, Figure 2 and Figure 4), the results show the importance of the sensor range. A range of only ±2 g (as in the case of MobiFall, MobiAct, Graz, Gravity Project, tFall or UniMiB SHAR datasets) may be a key limiting factor for a proper characterization of the user movements, as many sudden and intense movements (caused by ADLs or falls) can exceed this range easily. Figures illustrate that the median values of the statistics derived from the SVM in those testbeds with a low range have a tendency to concentrate near the full-scale input range of the variable (3.46 g if the maximum measured values of the acceleration components is 2 g). This ‘saturation’ is detected for the measurements of both ADLs and falls, so the range may clearly hamper the detection process. Conversely, those datasets (e.g., DLR, UMAFall, TST and SisFall) that employ a sensor with a higher range maximize the distance of the medians of falls and ADLs, while maximum detected values exhibit a different behavior for both groups of movements.

In any case, from the Figures we can also observe the extreme difficulty of discriminating falls from ADLs if just a threshold policy is going to be applied to these statistics. For most datasets, the boxes representing the distance between the 25th and 75th percentiles of falls and ADLs overlap partially. This overlapping zone is more evident for all datasets if we consider the intervals defined by the whiskers (as the interval obtained for the falls practically integrates the intervals computed for the ADLs). Again this confusion is more patent for those datasets using accelerometers with a lower range. Furthermore, Figures prove that an adequate election of any decision threshold is clearly determined by the characteristics of the employed sensor (no ‘natural’ acceleration threshold can be predefined with independence of the measurement system).

This difficulty for the identification of the movements is detected for the four analyzed statistics. As a matter of fact, from the visual inspection of the Figure 3 and Figure 4, no further improvement in the recognition seems to be achieved if those statistics resulting from the filtering of the acceleration (${\bar{SMV}}_{max}$ and $SMV_{d_{max}}$) are considered instead of utilizing the maximum and minimum values.

One important aspect that is normally neglected in many works on FDSs is the impact of the typology of the tested ADLs on the performance of the detector. Some studies offer a confusion matrix that describes the effectiveness of the FDS to recognize every considered type of non-fall activity as an ADL. However, the capability of the proposed FDS to discriminate a certain category of ADLs from falls is not usually assessed.

**Figure 5:**
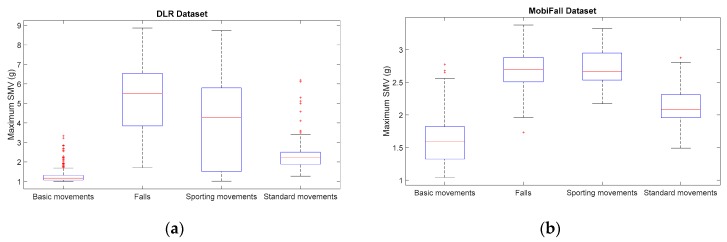

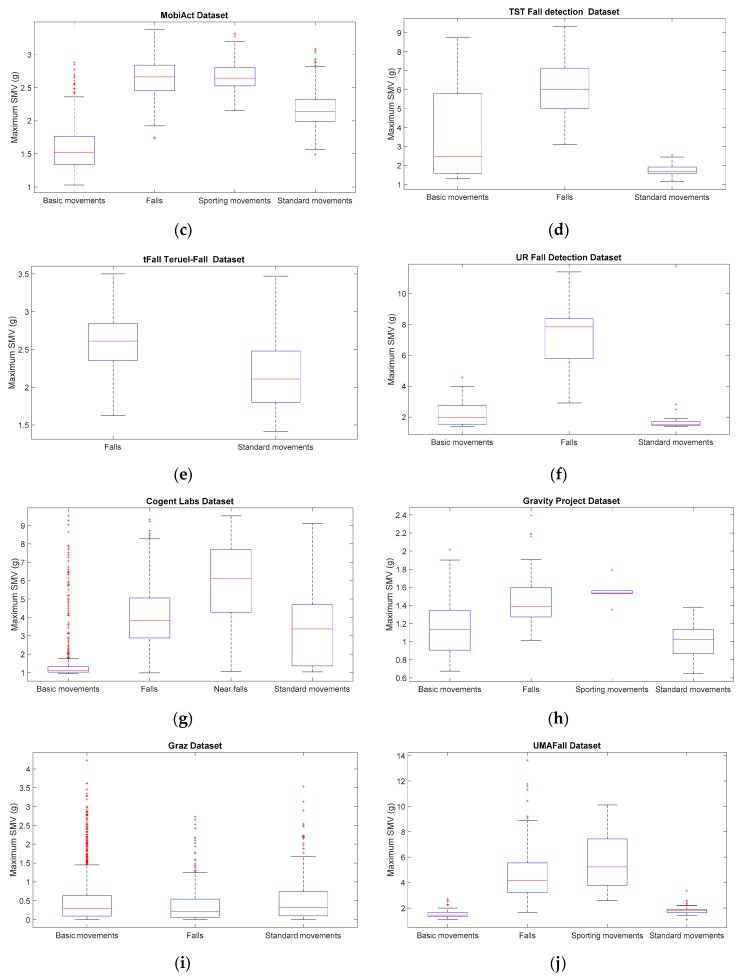

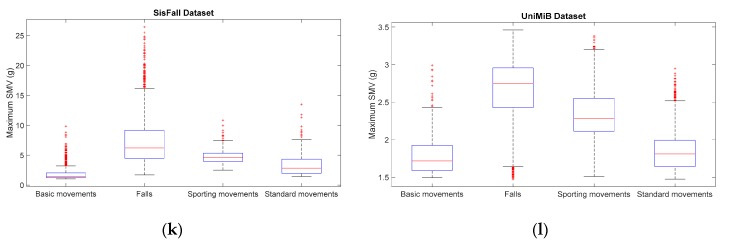
Boxplots of the of the maximum acceleration module for the falls and the three considered categories of ADL (for all the datasets only the acceleration signal at the waist or—if not available—at the thigh is considered). (**a**) DLR Dataset; (**b**) MobiFall Dataset; (**c**) Mobiact Dataset; (**d**) TST Fall Detection Dataset; (**e**) t-Fall Dataset; (**f**) UR Fall Detection Dataset; (**g**) Cogent Labs Dataset; (**h**) Gravity Project Dataset; (**i**) Graz Dataset; (**j**) UMAFall Dataset; (**k**) SisFall Dataset; (**l**) UniMiB SHAR Dataset.

**Figure 6:**
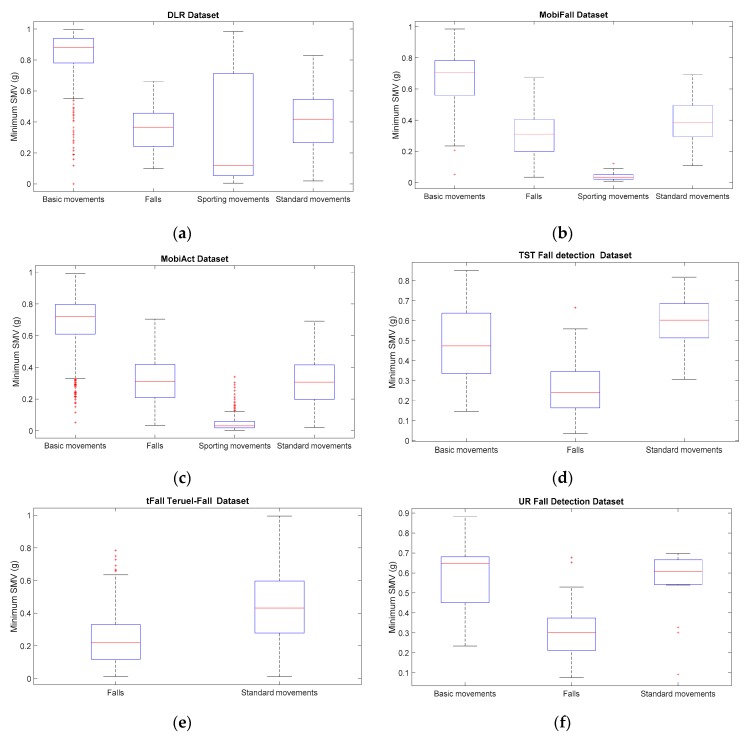

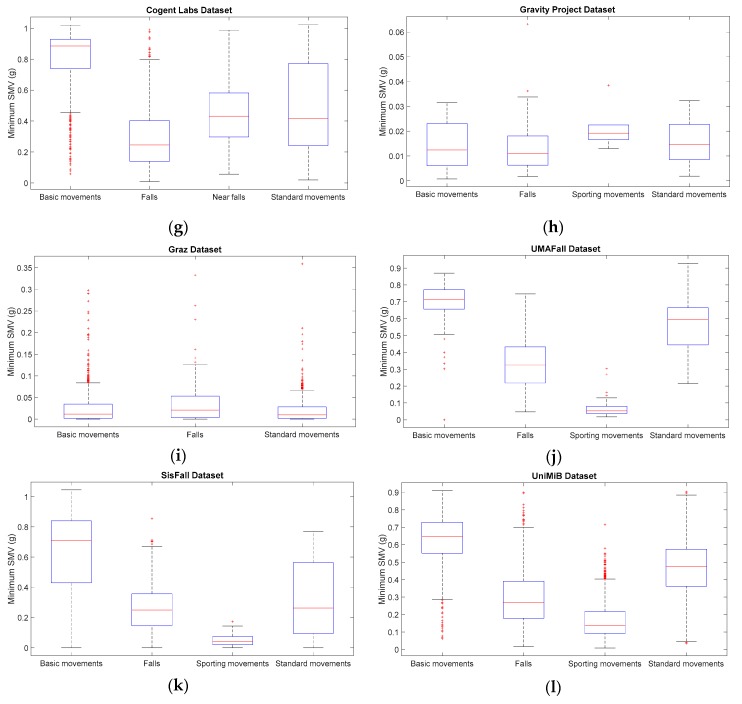
Boxplots of the of the minimum acceleration module for the falls and the three considered categories of ADL (for all the datasets only the acceleration signal at the waist or—if not available—at the thigh is considered). (**a**) DLR Dataset; (**b**) MobiFall Dataset; (**c**) Mobiact Dataset; (**d**) TST Fall Detection Dataset; (**e**) t-Fall Dataset; (**f**) UR Fall Detection Dataset; (**g**) Cogent Labs Dataset; (**h**) Gravity Project Dataset; (**i**) Graz Dataset; (**j**) UMAFall Dataset; (**k**) SisFall Dataset; (**l**) UniMiB SHAR Dataset.

Figure 5 and Figure 6 show the results of repeating the previous statistical analysis when the categorization of ADLs proposed in Table 6 is contemplated. In particular, the boxplots depicted in the Figures correspond to the maximum (Figure 5) and minimum (Figure 6) values of the SMV when they are independently computed for the three aforementioned categories of ADL (basic movements, standard movements) in the 12 published datasets. Very similar results (not included here) are obtained for the other two statistics (${\bar{SMV}}_{max}$ and $SMV_{d_{max}}$). In the case of tFall dataset (Figure 5c and Figure 6c), just a single category is displayed given that the traces (which were recorded during the daily life of the experimental subjects) do not include any description about the nature of every particular activity. As it could be expected, the Figures show the strong divergences of the statistics computed for the three ADL categories. The plots confirm that, in most cases, falls could be effortlessly discriminated from basic movements just by setting a simple detection threshold for the peak and the minimum values of the SMV. For the case of the standard activities (walk, climbing stairs, etc.) this behavior is also reported, especially in the datasets with more samples (DLR, MobiAct, SisFall or UniMiB SHAR). Conversely, the box plots related to the sporting activities visibly overlap with those corresponding to the fall movements. Moreover, in some datasets (e.g., Gravity project, UMAFall) the median of the peaks of the SMV caused of the sporting activities is higher than in the case of falls. Likewise, the near-falls, which were emulated in the Cogent dataset, also generate acceleration peaks higher than the falls themselves. A similar behavior is observed for the analysis of the free fall phase. As Figure 6 illustrates, except for the case of Gravity Project, in all the repositories that include sporting activities, the median of the minimum SMV that is detected for this sort of activity is lower than the same measured statistics for the case of falls.

These results evidence the need of particularizing the analysis of any FDS as a function of the nature of the ADLs with which the detection process is going to be validated.

## 8. Conclusions

This work has presented an exhaustive analysis of publicly available datasets intended for the research on wearable fall detection systems. After a thorough bibliographic search, twelve different datasets (based on the signals captured through the monitoring of a set of experimental subjects transporting one or several inertial sensors) were found and compared from different point of views.

In spite of the existence of recommendations to define these benchmarking datasets, the performed analysis uncovers a complete lack of consensus about the way in which the experimental testbeds are configured and documented. Thus, the systematic comparison of the datasets reveals a high heterogeneity in aspects such as the procedure to emulate the movements, the number of experimental subjects, the duration and number of tested movements, the format in which the samples are stored, the number, nature (smartphone or external IMU) and position of the sensors, etc. In this regard, the statistical study of the samples shows the remarkable impact of the sensor range (mainly the triaxial accelerometer, which is the unique common sensor that is considered in all the datasets). Many datasets employ an accelerometer with a range of ±2 g (19.62 m/s^2^), which cannot be sufficient for a proper discrimination of the acceleration peaks caused by the impacts provoked by falls. In fact, the detected range for the measurements of the acceleration modules varies enormously from one dataset to another. This indicates the difficulty of setting an “abstract” and invariant discrimination threshold that can be considered valid for any fall detection system. Conversely, any thresholding policy should strongly rely on the range of the employed sensors.

Besides, the assessment of the samples has verified the importance of classifying the different types of ADLs that are going to be employed in the evaluation of a FDS, as long as the discrimination of falls from those activities with a high degree of mobility may imply a very specific challenge.

Finally, to sum up, we recapitulate the main recommendations and conclusions that can be drown from the performed analysis:

- In our opinion, when a dataset is selected, the number of samples and experimental subjects, as well as the variety of tested types of ADLs and falls should be prioritized as selection criteria.
- The impact of the range of the employed sensors (especially the accelerometer) on the validity of the samples should not be neglected before the use of the samples for validation purposes.
- Given the heterogeneity of existing repositories, the efficacy of a FDS should be always contrasted against more than one dataset.
- The evaluation of a FDS should include datasets (such as tFall) which incorporate traces captured during the actual daily life of the experimental subjects.
- Apart from describing the particular movements emulated during the experiments, ADLs should be classified into a small number of general categories depending on the mobility of the performed activities. This categorization is required to assess the actual effectiveness of the FDS when applied to different target populations.
- Future datasets should insist on collecting data (at least from ADLs) from a population of older people. To this day, the influence of the age of the experimental subjects on the characteristics of the available datasets has not been studied in detail.
- A common procedure to generate and document fall datasets is still required.
- The lack of a public dataset with a representative number of samples obtained from real-life falls noticeably hinders the reliability of the research on FDS.

## Figures and Tables

**Table 1:** Basic characteristics of public datasets of falls and Activities of Daily Living (ADLs).

Dataset	Ref.	Authors	Institution	City (Country)	Year
DLR	[29]	Frank et al.	German Aerospace Center (DLR)	Munich (Germany)	2010
MobiFall MobiAct	[30] [31]	Vavoulas et al.	BMI Lab (Technological Educational Institute of Crete)	Heraklion (Greece)	2013 2016
TST Fall detection	[32]	Gasparrini et al.	TST Group (Universita Politecnica delle Marche)	Ancona (Italy)	2014
tFall	[33]	Medrano et al.	EduQTech (University of Zaragoza)	Teruel (Spain)	2014
UR Fall Detection	[34]	Kępski et al.	Interdisciplinary Centre for Computational Modelling (University of Rzeszow)	Krakow (Poland)	2014
Cogent Labs	[35]	Ojetola et al.	Cogent Labs (Coventry University)	Coventry (UK)	2015
Gravity Project	[36]	Vilarinho et al.	SINTEF ICT	Trondheim (Norway)	2015
Graz	[37]	Wertner et al.	Graz University of Technology	Graz (Austria)	2015
UMAFall	[38]	Casilari et al.	Dpto. Tecnología Electrónica (University of Málaga)	Málaga (Spain)	2016
SisFall	[39]	Sucerquia et al.	SISTEMIC. Univ. of Antioquia	Antioquia (Colombia)	2017
UniMiB SHAR	[40]	Micucci et al.	Department of Informatics, Systems and Communication (University of Milano)	Bicocca, Milan (Italy)	2017

**Table 2:** URL from which the traces can be downloaded and file format of the dataset.

Dataset	URL (Available on 28 March 2017)	File Type
DLR	http://www.dlr.de/kn/en/desktopdefault.aspx/tabid-8500/14564_read-36508/	2 Matlab files containing 1 matrix per subject and trial
MobiFall MobiAct	http://www.bmi.teicrete.gr/index.php/research/mobiact	1 text file (with comma-separated values) per subject, activity and trial
TST Fall detection	http://www.tlc.dii.univpm.it/blog/databases4kinect	1 binary file per subject, activity and trial
tFall	http://eduqtech.unizar.es/fall-adl-data/	1 file (extension .dat) with comma-separated values per subject, activity and trial
UR Fall Detection	http://fenix.univ.rzeszow.pl/~mkepski/ds/uf.html	1 CSV file per subject, activity and trial
Cogent Labs	http://cogentee.coventry.ac.uk/datasets/fall_adl_data.zip	1 text file (with comma-separated values) per subject including all the experiments
Gravity Project	https://github.com/SINTEF-SIT/project_gravity	1 json file per subject, activity and trial
Graz	https://figshare.com/articles/Dataset_for_Mobile_Phone_Sensing_Based_Fall_Detection/1444405	1 SQLite database with 13 tables
UMAFall	https://figshare.com/articles/UMA_ADL_FALL_Dataset_zip/4214283 http://webpersonal.uma.es/de/ECASILARI/Fall_ADL_Traces/UMA_FALL_ADL_dataset.html	1 CSV file per subject, activity and trial
SisFall	http://sistemic.udea.edu.co/en/investigacion/proyectos/english-falls/	1 text file (with comma-separated values) per subject and activity type (ADL or fall)
UniMiB SHAR	http://www.sal.disco.unimib.it/technologies/unimib-shar/	3 Matlab files with the traces, the activity label and name of the activity

**Table 3:** Characteristics of the experimental subjects.

Dataset	Number of Subjects (Female/Male)	Age (Years)		Weight (Kg)		Height (cm)	
		Range	μ ± σ	Range	μ ± σ	Range	μ ± σ
DLR ^1,2^	19 (8/11)	[23–52]	30 ± 7.66	n.i.	n.i.	[160–183]	171.67 ± 8.23
MobiFall	24 (7/17)	[22–47]	27.46 ± 5.26	[50–103]	76.42 ± 14.78	[160–189]	174.67 ± 7.51
MobiAct	57 (15/42)	[20–47]	25.26 ± 4.24	[50–120]	76.60 ± 14.54	[160–193]	175.39 ± 7.39
TST Fall detection	11 (n.i.)	[22–39]	n.i.	n.i.	n.i.	[162–197]	n.i.
tFall	10 (3/7)	[20–42]	31.30 ± 8.60	[54–98]	69.20 ± 13.1	[161–184]	173.00 ± 8
UR Fall Detection	6 (0/6) ^3^	n.i. (age over 26)		n.i.	n.i.	n.i.	n.i.
Cogent Labs	42 (6/36)	[18–51]	24.12 ± 5.72	[43–108]	69.71 ± 13.08	[150–187]	172.24 ± 6.76
Gravity Project	2 (n.i.) ^4^	[26–32]	29 ± 4.24	[63–80]	71.5 ± 12.02	[170–185]	177.50 ± 10.61
Graz	5 (n.i.)	n.i.	n.i.	n.i.	n.i.	n.i.	n.i.
UMAFall	17 (7/10)	[18–55]	26.71 ± 10.47	[50–93]	69.88 ± 12.68	[155–195]	171.53 ± 9.37
SisFall	38 (19/19)	[19–75]	40.16 ± 21.33	[41.5–102]	62.30 ± 12.63	[149–183]	164.05 ± 9.27
UniMiB SHAR	30 (24/6)	[18–60]	27 ± 11	[50–82]	64.40 ± 9.7	[160–190]	169.00 ± 7

n.i.: Not indicated in the documentation of the dataset; ^1^ The characteristics of one subject are not described; ^2^ Data also includes three extra subjects used only for tests; ^3^ Six male subjects were detected from the associated videos (although the documentation informs that the dataset was generated by five users); ^4^ Three participants are reported in the documentation but the traces just include the registered movements of two of them.

**Table 4:** Environment of the testbed and spontaneity of the movements.

Dataset	Scenario of the Experiment	Spontaneity of the Movements	Use of Mattress to Cushion the Falls	Video Clips
DLR	Real life indoor (meeting in an office, etc.) and outdoor scenarios (forest, bus stop)	Semi-naturalistic conditions	No	Yes
MobiFall MobiAct	Gym Hall Not described	Predefined	Yes (5 cm-thick mattress)	No
TST Fall detection	Office or lab	Predefined	Yes	Yes (blurred)
tFall	One week of everyday behavior (ADLs)	Naturalistic conditions (ADL) Predefined (falls)	Use of compensation strategies to prevent the fall	No
UR Fall Detection	Office & Home environment	Predefined	No	Yes
Cogent Labs	Office	Predefined ^1^	Yes	No
Gravity Project	Not commented	Predefined	Yes (1 cm fitline mattress, and soft 5.5 cm-thick mattress on top of a 3.5 cm martial arts mattress)	No
Graz	Gym Hall	Predefined	No (wooden floor)	No
UMAFall	Home environment	Predefined	Yes	Yes
SisFall	Gym Hall	Predefined	Yes	Yes
UniMiB SHAR	Not commented	Predefined	Not commented	No

^1^ Some falls were forced by pushing a blindfolded subject.

**Table 5:** Number, typology and duration of the samples in the datasets.

Dataset	Number of Types of ADLs/Falls	Number of Samples (ADLs/Falls)	Duration of the Samples (s)		
			[Minimum–Maximum]	Mean	Median
DLR	15/1	1017 (961/56)	[0.27–864.33] s	18.40 s	9.46 s
MobiFall	9/4	630 (342/288)	[0.27–864.33] s	18.40 s	9.46 s
MobiAct	9/4	2526 (1879/647)	[4.89–300.01] s	22.35 s	9.85 s
TST Fall detection	4/4	264 (132/132)	[3.84–18.34] s	8.60 s	8.02 s
tFall	Not typified/8	10,909 (9883/1026)	6 s (all samples)	6 s	6 s
UR Fall Detection	5/4	70 (40/30)	[2.11–13.57] s	5.95 s	5.256 s
Cogent Labs	8/6	1968 (1520/448)	[0.53–55.73] s	13.15 s	12.79 s
Project Gravity	7/12	117 (45/72)	[9.00–86.00] s	27.53 s	23.00 s
Graz	10/4	2460 (2240/220)	[0.18–961.23] s	12.67 s	4.32 s
UMAFall	8/3	531 (322/209)	15 s (all samples)	15 s	15 s
SisFall	19/15	4505 (2707/1798)	[9.99–179.99] s	17.60 s	14.99 s
UniMiB SHAR	9/8	7013 (5314/1699)	1 s (all samples)	1 s	1 s

**Table 6:** Types of the activities (ADLs and falls) executed by the experimental subjects.

Dataset			Activities of Daily Living (ADLs)		Near Falls	Falls	
	Simple Movements		Standard Normal Life Movements	Sporting Activities			
DLR	- Getting up - Going down - Lying - Sitting - Standing		- Walking - Walking downstairs - Walking upstairs	- Accelerating - Decelerating - Jumping - Jumping backward - Jumping forward - Jumping vertically - Running		- No particular type is defined	
MobiFall & MobiAct	- Sitting on a chair - Stepping in a car - Stepping out of a car - Standing		- Normal walking - Going downstairs - Going upstairs	- Jogging - Jumping		- Forwards (use of hands to dampen fall) - Forwards (first impact on knees) - Sideward bending legs - Backward (while trying to sit down)	
TST Fall detection	- Lying down on a mattress - Sitting on a chair		- Walking and grasp an object from the floor - Walking back and forth			- Backwards (end up lying) - Backwards (end up sitting) - Frontal fall (end up lying) - Fall to the side (end up lying)	
tFall			- ADLs are not emulated: users are monitored during their daily life			- Backwards - Fall while sitting on chair - Forwards - Forwards with protection strategies	- Hitting an obstacle in the fall - Lateral left fall - Lateral right fall - Syncope
UR Fall Detection	- Lying on the floor - Lying on the sofa - Sitting down		- Crouching down - Picking-up an object from the floor			- Forwards while seated - Forwards while walking	- Lateral fall while seated - Lateral fall while walking
Cogent Labs	- Standing - Lying - Sitting on a bed	- Sitting on a chair	- Walking - Crouching - Going downstairs and upstairs		- Near-fall	- Forwards - Backwards - Rightwards	- Leftwards - ‘Real’ forward fall ^1^ - ‘Real’ backward fall ^1^
Gravity Project	- Turning around - Sitting down slowly - Sitting down quickly - Riding an elevator		- Tying shoes - Walking - Going downstairs and upstairs	- Running		- Forwards (fainting with bent knees) - Forwards (while stepping down) - Self tripping - Backwards (with a round back and bent knees) - Backwards (while sitting) - Backwards (against a wall) - Backward (and turning to the left side) - Backward (and turning to the left right) - Leftwards (with bent knees) - Leftwards (landing at the base of a wall) - Rightwards (landing at the base of a wall) - Falling from a sitting position	
Graz	- Stopping - Standing - Sitting down slowly - Sitting down quickly - Getting up - Bending forward - Lying on the floor		- Going downstairs - Going upstairs - Walking			- Stumbling - Slipping - Sliding - Get unconscious	
UMAFall	- Bending - Lying down on a bed - Sitting on a chair & getting up		- Walking - Going downstairs - Going upstairs	- Hopping - Jogging		- Backwards - Forwards - Lateral fall	
SisFall	- Sitting down and getting up: (1) slowly from a half height chair (2) slowly from a low height chair (3) quickly from a half height chair (4) quickly from a low height chair - Sitting (from lying position) and vice versa: (1) slowly (2) quickly - Changing position while lying - Stepping in and out of a car - Bending at knees and getting up - Bending without bending knees and getting up		- Walking slowly - Walking quickly - Going upstairs and downstairs: (1) slowly (2) quickly	- Jogging slowly - Jogging quickly - Jumping (trying to reach an object)	- Stumbling while walking - Collapse into a chair while getting up	- Forward fall while walking caused by a slip - Backward fall while walking caused by a slip - Lateral fall while walking caused by a slip - Forward fall while walking caused by a trip - Forward fall while jogging caused by a trip - Vertical fall while walking caused by fainting - Fall while walking caused by fainting 5 15 s - Forward fall when trying to get up - Lateral fall when trying to get up - Forward fall when trying to sit down - Backward fall when trying to sit down - Lateral fall when trying to sit down - Forward fall while sitting, caused by fainting - Backward fall while sitting, caused by fainting - Lateral fall while sitting, caused by fainting	
UniMiB SHAR	- Sitting down on a chair - Getting up from a chair - Lying on a bed - Getting up from bed		- Walking - Going upstairs - Going downstairs	- Running - Jumping		- Forwards - Backwards - Leftwards - Rightwards - Falling with contact to an obstacle - Syncope - Falling while sitting down on a chair - Falling using compensation strategies to prevent the impact	

^1^ In these ‘real’ falls users stand on a wobble board while blindfolded and then pushed from behind or from the front to provoke a fall.

**Table 7:** Number, sensors, positions and measured magnitudes of the sensing points.

Dataset	Number of Sensing Points	Number of Magnitudes Recorded Per Sensing Point	Recorded Magnitudes	Positions of the Sensing Points
DLR	1	3	A, G, M	Waist (belt)
MobiFall & MobiAct	1	3	A, G, O	Thigh (trouser pocket)
TST Fall detection	2	1	A	Waist Right wrist
tFall	1	1	A	Alternatively: Thigh (right or left pocket) Hand bag (left or right side)
UR Fall Detection	1	1	A	Waist (near the pelvis)
Cogent Labs	2	2	A, G	Chest Thigh
Gravity Project	1 or 2 *	1	A	Thigh (smartphone in a pocket) Wrist (smartwatch)
Graz	1	2	A, O	Waist (belt bag)
UMAFall	5	3	A, G, M **	Ankle Chest Thigh (right trouser pocket) Waist Wrist
SisFall	1	3	A, A, G ***	Waist
UniMiB SHAR	1	1	A	Thigh (left or right trouser pocket)

Note: A: Accelerometer, G: Gyroscope, O: Orientation measurements, M: Magnetometer. * Only a group of samples contain the traces monitored by both the smartphone and the smartwatch. ** The smartphone only captured the acceleration signal. *** The employed sensing unit included two accelerometers from different vendors.

**Table 8:** Characteristics of the sensors.

Dataset	Type of Sensor	Model	Sampling Rate (Hz)	Presumed Range	Minimun & Maximum Values in the Traces	Resolution
DLR	1 external IMU	Xsens MTx (Enschede, The Netherlands)	100	±5 g (A) ±1200°/s (G) ±75 μT (M)	[−6.3958–6.5584] g [−14.35–15.39] °/s [−2.9179–3.0207] μT	16 bits (A) 16 bits (G) 16 bits (M)
MobiFall & MobiAct	1 smartphone	Samsung Galaxy S3 (Seul, Korea) (LSM330DLC IMU) (STMicroelectronics, Geneva, Switzerland)	87 (A) 100 (G,O)	±2 g (A) ±2000°/s (G) ±360° (O)	[−1.9951–1.999] g [−573.44–573.42] °/s [−179.9995–360] °	12 bits (A) ^1^ 16 bits (G) ^1^
TST Fall detection	2 external IMUs	Shimmer device (Dublin, Ireland)	100	±8 g (A)	[−5.4973, 5.5054] g	16 bits
tFall	1 smartphone	Samsung Galaxy Mini (Seul, Korea)	45 (±12)	±2 g (A)	[−2.0303–2.082] g	20 bits ^1^
UR Fall Detection	1 external IMU	x-io x-IMU (Bristol, UK)	256	±8 g (A)	[−8.0493–8.0191] g	12 bits
Cogent Labs	2 external IMUs	Shimmer device (Dublin, Ireland)	100	±8 g (A) ±2000°/s (G)	[−5.3279–5.8552] g [−714.02–721.71] °/s	16 bits 16 bits
Gravity Project	1 smartphone 1 smartwatch	Samsung Galaxy S3 (Seul, Korea) LG G Watch (Seul, Korea)	50	±2 g (A) ±16 g (A)	2.39 g (SVM) ^2^ 14.8 g (SMV) ^2^	36 bits ^1^
Graz	1 smartphone ^3^	Samsung Galaxy S5 (Seul, Korea) mini Samsung Galaxy S5 (Seul, Korea) Google Nexus 4 (LG, Seul, Korea) Google Nexus 5 (LG, Seul, Korea) Sony Xperia Z3 (Tokyo, Japan)	5	±2 g (A) ±360° (O)	[−2.2838–2.4655] g [−2.9463–2.9754] g [−2.4937–2.6053] g [−2.7741–2.7799] g [−2.3516–2.4218] g [−18.35–36.70] ° (all models)	36 bits ^1^
UMAFall	1 Smartphone ^4^ 4 external IMUs	Samsung Galaxy S5 (Seul, Korea) (InvenSense MPU-6500) LG G4 (Seul, Korea) (Bosch Accelerometer) (Gerlingen, Germany) Texas Instruments SensorTag (Dallas, TX, USA) (InveSense MPU-9250) (San Jose, CA, USA)	100 20	±2 g (A) ±16 g (A) ±8 g (A) ±256°/s (G) ±4800 μT (M)	[−1.9999–1.999] g [−4.0444–4.0378] g [−7.9373–8] g [−256.78–254.81] °/s [−190.83–100.67] μT	16 bits 16 bits 16 bits 16 bits 14 bits
SisFall	1 external sensing mote with two accelerometers (A1&A2) and a gyroscope	Self-developed prototype: A1: Analog Device ADXL345 (Norwood, MA, USA) A2: NXP MMA8451Q (Eindhoven, The Netherlands), ITG3200 gyroscope (Texas Instruments, Dallas, Texas, USA)	200	±16 g (A1) ±8 g (A2) ±2000°/s (G)	[−16–15.99] g [−8–7.999] g [−1971.7–1999.9] °/s	13 bits 14 bits 16 bits
UniMiB SHAR	1 smartphone	Samsung Galaxy Nexus (Seul, Korea)	50	±2 g (A)	[−2.0001–2.0001]	52 bits ^1^

Notes: A: Accelerometer, G: Gyroscope, O: Orientation measurements, M: Magnetometer, n.i.: not indicated in the dataset documentation. ^1^ Deduced from the maximum values and the minimum quantification step detected in the traces (not coherent with the IMU datasheets). ^2^ The traces only characterize the module of the acceleration and the vertical acceleration (not the individual components of the acceleration). ^3^ Each of the five experimental subjects utilized a different model of smartphone. ^4^ Two smartphones were alternatively utilized in the testbed.
